# EMAST is a Form of Microsatellite Instability That is Initiated by Inflammation and Modulates Colorectal Cancer Progression

**DOI:** 10.3390/genes6020185

**Published:** 2015-03-31

**Authors:** John M. Carethers, Minoru Koi, Stephanie S. Tseng-Rogenski

**Affiliations:** Division of Gastroenterology, Department of Internal Medicine, University of Michigan, 3101 Taubman Center, 1500 East Medical Center Drive, Ann Arbor, MI 48109, USA; E-Mails: mkoi@med.umich.edu (M.K.); sstseng@med.umich.edu (S.S.T.-R.)

**Keywords:** DNA mismatch repair, microsatellite instability, genomic instability, colorectal cancer, MSH3, MutSβ, inflammation, short tandem repeats, EMAST, patient survival, patient outcome

## Abstract

DNA mismatch repair (MMR) function is critical for correcting errors coincident with polymerase-driven DNA replication, and its proteins are frequent targets for inactivation (germline or somatic), generating a hypermutable tumor that drives cancer progression. The biomarker for defective DNA MMR is microsatellite instability-high (MSI-H), observed in ~15% of colorectal cancers, and defined by mono- and dinucleotide microsatellite frameshift mutations. MSI-H is highly correlated with loss of MMR protein expression, is commonly diploid, is often located in the right side of the colon, prognosticates good patient outcome, and predicts poor efficacy with 5-fluorouracil treatment. Elevated microsatellite alterations at selected tetranucleotide repeats (EMAST) is another form of MSI at tetranucleotide repeats that has been observed in multiple cancers, but its etiology and clinical relevance to patient care has only been recently illuminated. Specifically, EMAST is an acquired somatic defect observed in up to 60% of colorectal cancers and caused by unique dysfunction of the DNA MMR protein MSH3 (and its DNA MMR complex MutSβ, a heterodimer of MSH2-MSH3), and in particular a loss-of-function phenotype due to a reversible shift from its normal nuclear location into the cytosol in response to oxidative stress and the pro-inflammatory cytokine interleukin-6. Tumor hypoxia may also be a contributor. Patients with EMAST colorectal cancers show diminished prognosis compared to patients without the presence of EMAST in their cancer. In addition to defective DNA MMR recognized by tetranucleotide (and di- and tri-nucleotide) frameshifts, loss of MSH3 also contributes to homologous recombination-mediated repair of DNA double stranded breaks, indicating the MSH3 dysfunction is a complex defect for cancer cells that generates not only EMAST but also may contribute to chromosomal instability and aneuploidy. Areas for future investigation for this most common DNA MMR defect among colorectal cancers include relationships between EMAST and chemotherapy response, patient outcome with aneuploid changes in colorectal cancers, target gene mutation analysis, and mechanisms related to inflammation-induced compartmentalization and inactivation for MSH3.

## 1. Introduction: DNA Mismatch Repair, Microsatellite Instability, and EMAST

The alteration of human DNA microsatellite sequences was first recognized among colorectal cancers (CRC) in 1993 [1,2,3]. Shortly thereafter, with realization that bacteria and yeast microsatellite frameshift mutations were caused by a defect in DNA mismatch repair (MMR) function, successful efforts identifying the human counterparts to the bacteria and yeast DNA MMR genes were undertaken. Those efforts demonstrated that mutations in DNA MMR within the germline was associated with a form of hereditary cancer now termed Lynch syndrome, where affected patients share an extremely high risk for CRC and other cancers of the female reproductive track, gastrointestinal track, and urological track [4,5]. Furthermore, some sporadic colorectal cancers were shown to have defective DNA MMR caused by hypermethylation of the promoter of the DNA MMR gene *MLH1*, preventing transcription of *MLH1* [5]. However, testing for microsatellite alterations among cancers and other conditions was initially haphazard until a standard definition was put in place through an National Cancer Institute-sponsored workshop, allowing comparisons to take place between studies that occurred thereafter [6]. That definition requires use of a panel of mono- and dinucleotide microsatellite markers that are strongly associated with loss of DNA MMR protein expression, and can identify colorectal cancers from patients that might influence their outcome and response to chemotherapy [6,7,8].

The human DNA MMR system is comprised of several proteins that interact as heterodimers to function for repair. MLH1 and MSH2 are common heterodimer partners to other MMR proteins (e.g., MutSα, a heterodimer of MSH2-MSH6, MutSβ, a heterodimer of MSH2-MSH3, and MutLα, a heterodimer of MLH1-PMS2) that allow MMR function within the nucleus and control of its protein stability, and both of which when absent completely abrogate DNA MMR function [9,10,11]. The recognition fidelity for DNA MMR lies with the MutS complexes that bind to DNA, with MutSα recognizing single bases-base mispairs and single insertion-deletion (I/D) loops, and MutSβ recognizing larger I/D loops [9,10] (Table 1). Functional overlap for repair between MutSα and MutSβ occurs at I/D loops of 2, or dinucleotide microsatellites (Table 2). Both *MLH1* and *MSH2* are the most common targets for mutation in the germline of Lynch syndrome patients, which completely abrogates DNA MMR function. Germline mutation of *MSH6*, a component of MutSα, causes a more moderate Lynch syndrome phenotype presumably due to overlap with MSH3 (MutSβ function) coupled with a compensatory increase in MSH3 expression [5,11]. *MSH6*-mutant carriers present at older ages than patients with *MLH1* or *MSH2* germline mutations [12]. Germline mutation of *PMS2*, a component of MutLα, is relatively rare [4,5]. There has been no description of a germline *MSH3* mutation as a cause of Lynch syndrome [5].

**Table 1:** Spectrum of recognition/repair function for the two MutS DNA mismatch repair recognition complexes. DSBs = double strand breaks.

	MutSα (MSH2-MSH6)	MutSβ (MSH2-MSH3)
Single mispaired nucleotides	Yes	No
Insertion-Deletion Loops		
1	Yes	No
2	Yes	Yes
3	No	Yes
4	No	Yes
5-fluorodeoxyuracil	Yes	Yes
O^6^-methylguanine adduct	Yes	No
6-thioguanine adduct	Yes	No
Cisplatin, carboplatin	Yes	No data, but triggers DSBs
Oxaliplatin, teraplatin, transplatin, JM335, JM216	No	No, but triggers DSBs
Irinotecan (CPT-11)	No	No

**Table 2:** Distribution of intrinsically-generated genomic DNA frameshift mutations among four different colorectal cancer cell lines with varying DNA mismatch repair-deficient backgrounds. Cells were subcloned, and obtained DNA from the cells were subcloned using TA cloning and sequenced for microsatellite instability at the genetic loci indicated. The total number of subclones is in the denominator, and the number of mutant subclones is in the numerator. I/D = insertion-deletion loop, with the number of nucleotides forming the loop.

		I/D = 1		I/D = 2		I/D = 3	I/D = 4		
CELL LINE	MMR-Status	BAT 25	BAT 26	D5S346	D17S250	TBP	RB	REN	HPRTII
SW480	Proficient	0/58 (0%)	0/58 (0%)	0/58 (0%)	0/58 (0%)	0/58 (0%)	0/58 (0%)	0/58 (0%)	0/58 (0%)
HCT 116	MLH1^−/−^ and MSH3^−/−^	26/46 (57%)	17/53 (32%)	13/47 (28%)	18/38 (47%)	13/83 (16%)	7/51 (14%)	23/91 (25%)	14/50 (28%)
HCT 116 + 3	MSH3^−/−^	0/111 (0%)	0/107 (0%)	3/102 (2.94%)	50/117 (43%)	6/53 (11%)	11/100 (11%)	29/52 (56%)	11/111 (10%)
DLD1	MSH6^−/−^	21/59 (36%)	15/58 (26%)	12/60 (20%)	26/71 (37%)	0/60 (0%)	0/80 (0%)	0/67 (0%)	0/59 (0%)

**Figure 1:**
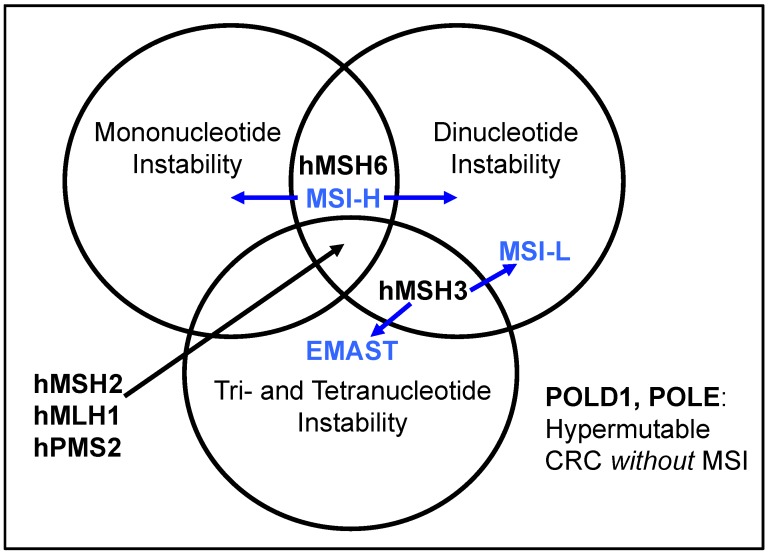
Spectrum of microsatellite frameshift mutations based on DNA mismatch repair protein function. Loss of function of MSH3 encompasses EMAST and MSI-L. Mutation of the DNA polymerases, *POLD1* and *POLE*, are found in hypermutable tumors but do not demonstrate microsatellite instability.

The phenomenon of elevated microsatellite alterations at selected tetranucleotide repeats (EMAST) has been observed among some cancers for two to three decades, paralleling the findings of frameshift mutations at mono- and dinucelotide repeats [13]. The cause for EMAST was elusive despite its observation, likely because (a) it did not involve MSH2 or MLH1, the two major DNA MMR proteins; (b) tools to study MSH6 and in particular MSH3 lagged behind those developed for MLH1 and MSH2; (c) there was no germline mutation detected for *MSH3* as a cause for Lynch syndrome, rendering it less important at least initially for this syndrome [14]; and (d) no connection could be made between somatic *MSH3* mutations and EMAST as *MSH3* frameshift mutations are observed in microsatellite instability-high (MSH-H) tumors, which already have complete deficiency of DNA MMR. Additionally, as a result of the NCI-defined panel for microsatellite alterations, microsatellite instability-low (MSI-L) was delineated and has been described in multiple tumors and inflammatory conditions, but had no clear connection with defective DNA MMR [6,15]. MSI-L tumors have been commonly lumped together with microsatellite stable (MSS) cancers because of no prior connection to defective DNA MMR [7,9,10]. Because the majority of MSI-L tumors show dinucleotide instability rather than mononucleotide instability (mononucleotides are most sensitive to frameshift mutation with MLH1, MSH2, and MSH6 deficiency), the suggestion has been made that the observation of EMAST and MSI-L might be one in the same, with the same etiology (Figure 1) [16,17,18,19].

## 2. Defining EMAST, and Its Overlap with MSI-L

EMAST is present when tetranucelotide microsatellite frameshift mutations occur in assayed human tissue, compared to control normal tissue. However, unlike the NCI consensus regarding the definition for MSI-H by clearly outlining the number of mono- and dinucleutide markers used and its strong association with loss of DNA MMR protein expression [6], there has been no official consensus to date regarding the definition of EMAST. Published papers have utilized one tetranucleotide marker mutated out of five or more markers as a definition [16,17,19,20], whereas others have utilized two tetranucleotide markers mutated to define the presence of EMAST [20,21,22,23]. Until a consensus panel convenes, this may be an open question for this field of study.

**Figure 2:**
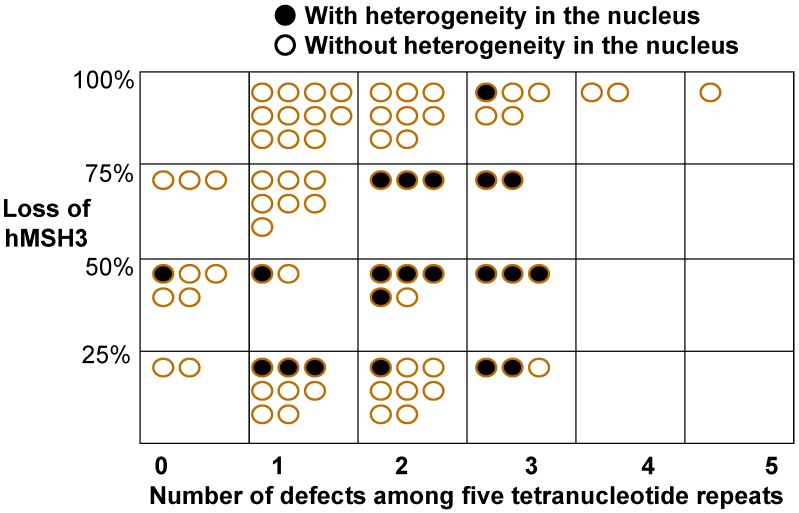
The degree of MSH3 protein loss matched with the number of tetranucleotide frameshift mutations. Filled-in circles represent colorectal cancers with nuclear expression heterogeneity, and open circles represents cancers without nuclear heterogeneity. From Lee S-Y, Chung H, Devaraj B, Iwaizumi M, Han HS, Hwang DY, Seong MK, Jung BH, Carethers JM. Elevated microsatellite alterations at selected tetranucleotide repeats are associated with morphologies of colorectal neoplasia [22].

Because of the association of EMAST with heterogeneous expression of the DNA MMR protein MSH3 (see below), an attempt to link the number of mutated tetranucleotide markers to MSH3 expression was made [22], much like the NCI consensus of MSI-H and DNA MMR protein expression among colorectal cancers [6]. In that study, five tetranucleotide repeats were used (*MYCL1, D9S242, D20S85, D8S321, and D20S82*) and among them, the highest correlation between tetranucleotide frameshift mutation and loss of MSH3 expression, particularly its nuclear heterogeneity, was when three tetranucleotide markers were mutated [22]. However, only three of 78 tumors showed more than three tetranuclueotide marker mutations, whereas the majority of tumors showed one, two or three markers mutated [22] (Figure 2). Most tumors with loss of MSH3 had one marker mutated (28/78, 36%) followed by two markers mutated (24/78, 31%) and three markers mutated (13/78, 17%) [22]. On the contrary, nuclear heterogeneity for MSH3, in addition to loss of MSH3 expression, correlated well with increasing number of tetranucleotide repeat mutations, from one tetranucleotide repeat to three repeats [22]. Overall, it is likely that both of these factors, MSH3 expression and MSH3 nuclear heterogeneity, are important for the strongest correlation between EMAST and MSH3 expression. A larger study is needed, along with a consensus among experts to help define the number of markers for studies.

Other complicating factors could be polymorphic differences among tetranucleotide markers, which may be less diverse for mutation compared with mono- or dinucleotide repeats, although this is not proven. For instance, among rectal tumors, the frequency of mutation of *MYCL1, D9S242, D20S85, D8S321, and D20S82* among EMAST tumors varies from 10%–65% [21]. Thus, the selection of markers may influence the frequency of frameshift and detection of EMAST, particularly if one uses only one marker positive for frameshift mutation as the definition. Additionally, mutant *TP53* status has been associated with EMAST in non-melanoma skin, bladder, and non-small cell lung cancers, which may influence the detection of EMAST [24,25]. This has not been demonstrated for colorectal cancers.

The NCI consensus workshop on microsatellite instability defined MSI-L as only one marker positive among a panel of mono- and dinucleotide markers [6]. Since that definition, multiple observations indicate that MSI-L is most often seen with frameshift mutation among dinucleotide repeats, a lack of association with *MSH6* mutation or loss [15], a lack of association with MSH2 or MLH1 loss, an association with inflammation, and is observed among EMAST cancers [16,17]. General consensus and evidence points that MSI-L and EMAST are observations that are caused by the same phenomenon of MSH3 deficiency (see Figure 1) [16,26,27]. Indeed, combining both MSI-L and EMAST can characterize a group of tumors that demonstrate poor prognosis among patients with CRC that is more powerful than either marker alone [19].

## 3. EMAST is a Biomarker Observed in Several Cancers and in Inflamed Non-Cancer Tissue

EMAST has been observed among cancers as a biomarker for two to three decades, although the term EMAST was not originally used. For instance, tetranucleotide microsatellite changes matching those in primary bladder tumors were detected in the urine from 19 of 20 (95%) patients, compared to only nine of 18 (50%) patients showing cancer cells from urine cytology [13], making tetranucleotide microsatellite instability a reliable biomarker for this tumor. EMAST has been reported in multiple solid organ malignancies in addition to bladder cancer, including: lung cancer, ovarian cancer, prostate cancer, renal cancer, endometrial cancer, non-melanoma skin cancer, head and neck cancers, and colorectal cancer (reviewed in [20]). The prevalence of EMAST among these cancers vary widely, ranging from 9%–75% [20]. This could be due to the type of tissue, as well as the number of tetranucleotide markers used for each study, for which most studies defined EMAST as one tetranucleotide marker showing frameshift mutation, with some utilizing two markers mutated [20]. Although EMAST has been identified among these tumors, its role as a biomarker for prognostication or any other utility for patient care has been poorly studied.

Among colorectal cancers, EMAST shows a strong correlation with the level of chronic inflammation in the tumor, in addition to its correlation with heterogeneous and decreased MSH3 expression [16,21,22,23]. Morphologically, EMAST was more likely found in downward-growing and ulcerated (depressed or excavated) colorectal cancers compared to sessile (superficial or flat) cancers and protruded (elevated or polypoid) cancers [22]. Microscopically, EMAST cancers show strong correlation with chronic inflammation, particularly immune cells within the glandular epithelium and in the surrounding stroma as epithelial cell nests [21]. Immune cells along the invasive margin of the cancer were not associated with EMAST. These findings suggested that there may be a connection between the close proximity of immune cells with the epithelial components of the tumor and the observation of EMAST. Indeed, EMAST colorectal tumors show higher density of CD8^+^ T cells, but not CD4^+^ T cells, in the surrounding tumor nest stroma compared to EMAST-negative tumors [23]. Additionally, the density of CD8^+^ T cells increased with adenoma-to-carcinoma progression, mirroring the increase in EMAST observance found during this histological advancement [22,23]. Other components of the inflammatory milieu need to be studied to further understand the connection between EMAST and immune cells.

Non-cancerous human specimens, such as pancreatitis (detected from pancreas juice and tissue) and ulcerative colitis (tissue), situations that would demonstrate acute and chronic inflammation within tissue, have showed evidence of MSI [28,29]. These studies were performed prior to the NCI consensus panel definition of MSI, defining MSI-H, MSI-L, and MSS [6]. In retrospect, these studies found dinucleotide microsatellite instability within these non-cancer but inflamed tissues [28,29], and applying current NCI consensus definitions, most samples would be reclassified as MSI-L. Given the association of MSI-L (defined by dinucleotide instability among the NCI consensus markers) and its correlation with EMAST (defined by tetranucleotide instability) and changes in MSH3 expression, these studies likely described EMAST among these inflamed tissues (although these studies did not examine MSH3 expression). This implies that the interaction of inflammation with the assayed epithelium may be the driver for EMAST. As stated above, morphological features of ulceration that is associated with increased local inflammation was more likely to associate with EMAST (as well as decreased MSH3 expression) [22]. Adenomas, which are neoplastic but not malignant lesions, can demonstrate EMAST, particularly those with ulceration or CD8^+^ T cell infiltration [22,23]. Benign familial hamartomatous polyps, which histologically demonstrate cystic epithelium surrounded by an inflammatory lamina propria, show EMAST and loss of MSH3 expression, further demonstrating that nondysplastic epithelium linked with inflammation associates with EMAST [30]. Non-transformed human colonic epithelial cells exposed to the pro-inflammatory cytokine IL-6 (see below) also demonstrate EMAST with a change in MSH3 nuclear expression [31]. All of these findings indicate a strong connection between inflammation, MSH3 altered expression, and EMAST, even in the absence of neoplastic transformation.

## 4. EMAST Occurs in Colorectal Cancer and Modifies Patient Outcome

EMAST was first described in colorectal cancers in 2008 [16]. EMAST has proven to be a very common finding among colorectal cancers, a finding more widespread than MSI due to hypermethylation of *MLH1* that is seen in ~15% of all colorectal cancers. Among cohorts examined, EMAST is present among 50%–60% of all colorectal cancers [16,17,22,23], and 33% among rectal cancers [21]. Therefore, EMAST represents the most common DNA mismatch repair defect found in colorectal cancers.

There is emerging evidence that this biomarker, EMAST, influences the survival outcome of patients. Among 147 patients with rectal cancer, EMAST correlated with stage III/IV patients compared with stage I/II patients (62% *vs.* 37%, *p* = 0.02) [21]. This suggests that EMAST may be more associated with advanced stage, meaning that it might be a contributor to poor outcomes among rectal cancer patients. This is strongly supported by a separate study of 167 patients with stage II/III colorectal cancer in which MSI-L/EMAST status was compared to MSI-H and highly microsatellite stable patients [19]. MSI-L/EMAST colorectal cancer patients demonstrated the worse recurrence-free survival among the three groups, and distant metastasis was more likely in this group, and was an independent predictor of recurrent metastasis from stage II/III colorectal cancer (Hazard Ratio 1.83, range 1.06–3.15, *p* = 0.03) [19]. Thus, the presence of EMAST appears to be a significant poor prognosticator for patients with colorectal cancer.

EMAST is also observed during the histological advancement of neoplasia in the colon, with prevalence increasing from well-differentiated adenocarcinomas (frequency of 12.5%) to moderately or poorly-differentiated adenocarcinomas (56.9% and 40%, respectively) [22]. This might reflect the increased level of inflammation during histological progression.

An additional association for EMAST is patient race. Rectal cancers from African Americans were more likely to demonstrate EMAST compared with Caucasian patients (49% *vs.* 26%, *p* = 0.014) [21]. Given that EMAST in colorectal cancers is associated with advanced stage and portends a poor prognosis, the higher prevalence among African American patients might be contributory to the overall higher morbidity and mortality in this racial group. This data is in addition to reduced prevalence of the good prognosticator MSI-H among African American patients with colorectal cancer compared with Caucasians (7% *vs.* 14%, *p* = 0.009) [32].

## 5. A Defect in the DNA Mismatch Repair Protein MSH3 is a Cause of EMAST

Based on bacteria and yeast data for DNA mismatch repair, defects in MSH3 (part of the MutSβ complex) for human tetranucleotide frameshift mutations might seem obvious, but this was not proven in humans until relatively recently. As mentioned above, molecular tools to examine MSH3 lagged behind those for MLH1 and MSH2 as one reason. Haugen *et al*. experimentally linked the first connection between EMAST and MSH3 in human colon cancer, showing: (a) loss of expression of MSH3 in colorectal cancers with EMAST; and (b) that MSH3-deficient cells (deficient background or through knockdown of *MSH3*) exhibited dinucleotide or greater microsatellite frameshift mutation [16]. Lee *et al*. further connected the number of tetranucleotide frameshifts with loss of MSH3 nuclear expression in colorectal cancers [22].

Data from two additional papers show convincingly that defects in MSH3 drive EMAST with the use of reporter plasmids. Tseng-Rogenski *et al*. created human colon cancer cells permanently transfected with plasmids containing the human tetranucleotide microsatellite loci *D8S321* [AAAG_12_] or *D20S82* [AAAG_16_] by which frameshift mutation would trigger enhanced green fluorescent protein expression [26]. By transfecting into colon cancer cells with various DNA mismatch repair backgrounds or by knockdown of *MSH3*, the authors demonstrate that MSH3 loss is responsible for ongoing tetranucleotide frameshifts, with rates of ~18 × 10^−4^ to 34 × 10^−4^ mutations/cell/generation, compared to *MSH6*-deficient cells at rates of ~0.8 × 10^−4^ mutations/cell/generation [26]. Although these experiments were designed to detect deletion of one microsatellite repeat unit, an important observation from the sequencing of clones after mutation was that both contraction and expansion frameshifts of the microsatellite occurred with *MSH3*-deficiency [26]. Congruent with this data, Campregher *et al*. showed with the use of [AAAG_17_] and [CA_13_] reporter plasmids that the presence of MSH3 resulted in increased stability at the tetranucleotide sequence and increased but only partial stability at the dinucleotide sequence [27]. Additionally, they showed that both expansion and contraction of the tetranucleotide sequence occurred in clones. Both papers data on tetranucleotide frameshifts from *MSH3* dysfunction contrast the near uniform observation that mono- or dinucleotide microsatellite sequences in human colorectal cancer only contract in size. Further examination of this phenomenon and why this occurs is under investigation.

An important concept is that *MSH3* inactivation does not appear to initiate oncogenic transformation. Through targeted silencing of *MSH3* in HCEC cells, Campregher *et al*. showed significant changes in 202 proteins that affect several fundamental cellular pathways, but none of them oncogenic. This was further supported by lack of colony growth of knock down cells in soft agar assays [27]. Rather, evidence suggests that MSH3 and its biomarker EMAST may be associated with modification of cancer behavior compared to initiating it.

An association between the presence of mutant *TP53* and EMAST has been made in non-melanoma skin, bladder, and non-small cell lung cancer specimens, particularly for non-invasive disease [24,25]. This raises the possibility of mutant *TP53* influencing the formation of tetranucleotide frameshifts, but this has not been further proven, nor shown for colorectal cancers.

## 6. A Driver for MSH3 Dysfunction and EMAST Appears to Be Oxidative Stress and Inflammation-Induced Cytokines, and Potentially Hypoxia

Until recently, there was no good explanation for the mechanism for loss of *MSH3* function in the cause of EMAST. Aside from secondary mutation of *MSH3* in the setting of an MSI-H colorectal cancer caused by frameshift of its [A_8_] microsatellite, there has been no example in the literature for: (a) germline mutation for *MSH3* for which to study its consequences; (b) evidence for somatic inactivation of *MSH3* (aside from MSI-H cancers) for which MSH3 function could be lost; or (c) epigenetic inactivation of MSH3 (such as that seen for *MLH1*).

Some clues for how MSH3 might be inactivated emerged in publications. Given the lack of evidence for mutation and epigenic inactivation, the defect for *MSH3* to cause EMAST had to be an acquired trait for the cancer, and commensurate with this idea is the increased prevalence of EMAST along the adenoma-to-carcinoma continuum. Additionally, MSH3 expression in EMAST colorectal cancers became heterogeneous among cells within the tumor, and the nuclei of the cancer cells themselves became heterogeneous for MSH3 expression [16,21,22,23]. Another key finding was the strong association of EMAST and inflammation (as well as MSI-L with inflammation) as outlined above. The congruence of inflammation and heterogeneous expression of MSH3 in EMAST colorectal cancers suggested that they might be related to each other. The inflammatory milieu of a colorectal cancer will contain free oxygen radicals from oxidative stress, inflammatory cytokines, and may be in a hypoxic and low pH environment.

Direct oxidative stress can impair DNA mismatch repair function. Using non-toxic levels hydrogen peroxide to simulate oxidative stress, Chang *et al*. demonstrated evidence for degradation of the steady-state levels of MSH6 and PMS2, but with no effect on MSH2 or MLH1 proteins 24 h post treatment [33]. The authors were unable to determine any effect on MSH3 protein levels, but experiments using recombinant MutSβ to complement hydrogen peroxide treated cell extracts for repair of two extra helical bases suggested a defect for MutSβ function [33]. Cell sensitivity to oxidative stress varies based on the status of DNA mismatch repair, with mismatch repair deficient cells being more sensitive to hydrogen peroxide compared to mismatch repair proficient cells [34]. Mismatch repair deficient mice demonstrate increased susceptibility to oxidative stress-induced intestinal cancers, suggesting that intact DNA mismatch repair simultaneously protects against mutagenesis and suppresses tumorigenesis induced by oxidative stress [35].

Tseng-Rogenski *et al*. examined what was happening to MSH3 protein within colorectal cancer cells using the hydrogen peroxide model. Overall, no reduction in MSH3 protein levels was detected in total cell extracts after hydrogen peroxide treatment [26,31]. However, using immunofluorescence microscopy, a striking intracellular location shift of MSH3 occurred, with MSH3 vacating its predominant location in the nucleus (where it is used for DNA repair) and relocating to the cytosol away from nuclear DNA [26]. This shift was detected as soon as 2 h after hydrogen peroxide treatment and peaked between 4 h and 8 h after treatment, with MSH3 returning to its nuclear location thereafter [26]. Thus, oxidative stress triggered a shift of location, which could lead to a loss of function phenotype for MSH3. This observation would completely explain the prior observation of heterogeneous nuclear expression of MSH3 in EMAST cancers. No other DNA mismatch repair proteins (MSH6, MLH1, MSH2) shifted location with hydrogen peroxide.

**Figure 3:**
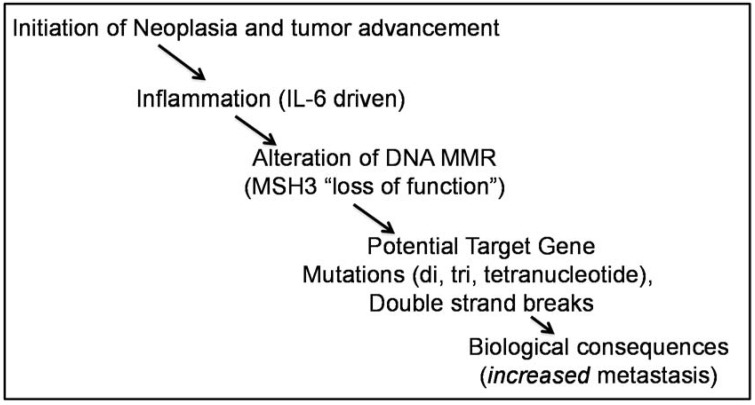
A model for modulation of the pathogenesis of colorectal cancer by EMAST. After the tumor has initiated, inflammation can modify the DNA repair function within the tumor through cytokine signaling, hypoxia, and oxidative stress. Evidence supports that Interleukin-6 can shift MSH3 protein from its nuclear locale to the cytosol, allowing accumulation of mutations and double strand breaks. It is believed that these genetic changes modify the tumor behavior, as patients with EMAST cancers present with advanced stage and are more likely to have metastasis.

Oxidative stress could be generated from a number of sources within a colorectal cancer. Tseng-Rogenski *et al*. examined pro-inflammatory cytokines as a potential source. After ruling out TNFα, IL1β, IFNα and IFNγ, the authors show that IL6 induces the MSH3 nuclear-to-cytosol compartmental shift, and is coincident with the generation of oxidative stress within colorectal cancer cells and non-transformed colon cells [31]. The MSH3 shift is dependent on IL6 trans-signaling through its soluble IL6 receptor and phosphorylation of STAT3 [31]. Mutations at genomic tetranucleotide loci were detected within two weeks in cells under IL6 treatment. Additionally, the authors show a strong correlation between IL6 presence in the colorectal cancer and EMAST [31]. These data indicate that the pro-inflammatory cytokine IL6 may be responsible for EMAST. The novel mechanism of mis-compartmentalization to inactivate MSH3 function in human cells is unique, and does not alter anything at the genetic or epigenetic level. In murine cells, MSH3 is a nuclear protein with a fine granular nucleoplasmic distribution and absent from condensed heterochromatin [36]. Upon ethanol or hydrogen peroxide treatment, murine MSH3 redistributed into nuclear bodies containing PCNA [36]. Overall, these observations further tie together previous findings of the convergence of inflammation, oxidative stress, MSH3 heterogeneous expression, and EMAST. With evidence that EMAST can worsen patient outcome from colorectal cancer, reducing the cause of EMAST appears to be a fruitful area in which may have a positive impact on patient care. Areas that might be targeted could be the inflammation itself, the IL6 signaling pathway, or the shuttling mechanism for MSH3, which at this time is not understood. One potential model for colorectal cancer based on the above information is presented in Figure 3.

Hypoxia and cellular pH changes can also alter DNA mismatch repair function. In particular, hypoxia and low extracellular pH reduces *MLH1* expression [37,38,39,40], apparently via hypoxia-induced transcription repressors and decreased histone methylation at the *MLH1* promoter [37,40,41]. Because PMS2 stability is dependent on association with its heterodimer partner MLH1, loss of MLH1 protein destabilizes PMS2 [11,39]. Although some manuscripts suggest little or no change in *MSH2* or *MSH6* expression with hypoxia [39], other manuscripts indicate that the transcription factor HIF-1α can displace other transcription factors from the *MSH2* promoter, reducing its expression (as well as subsequent stability of its heterodimer partner MSH6) [42]. Within murine and human stem cells, HIF-1α positively regulated *MLH1* and *MSH6* expression with short-term hypoxia, but prolonged hypoxia reduced both *MLH1* and *MSH6* expression through epigenetic regulation of these two gene promoters [43]. Overall, hypoxia and its accompanying low pH can enrich for mismatch repair deficient cells and generate drug-resistant clones in the remaining surviving population [44]. Regarding EMAST and *MSH3* regulation, Li *et al*., demonstrated that hypoxia induced a HIF-1α complex that could bind to two putative hypoxia response elements in the *MSH3* promoter to reduce *MSH3* expression [45]. These data need to be further explored in human colorectal cancers.

## 7. Additional Considerations for Pathogenesis of Colorectal Cancer by MSH3 Dysfunction

Unlike MSI-H colorectal cancers in which hypermethylation of *MLH1* drives multiple target gene mutations, to date there has been little evidence for mutation of specific target genes that might drive or alter the pathogenesis of colorectal cancer with loss of MSH3 function. One study suggested that there are 10 human genes with tetranucleotide coding microsatellites reported, with none of them frameshifted in EMAST bladder cancers, although colorectal cancers were not examined [46]. However, that study might underestimate the number of tetranucleotide coding microsatellites given current modern sequencing technologies. Several genes have trinucleotide coding repeats that might be capable of undergoing frameshift mutation, but again, there is little evidence that this occurs and contributes to colorectal cancer pathogenesis. One group found an association among MSI-H colorectal cancers with frameshift mutations in the transcription factor *E2F4* [CAG_13_] and secondary frameshift mutations of *MSH3* [A_8_] (see below) [47]. *E2F4* frameshift mutations have previously been found among MSI-H colorectal cancers [48]. At present, it is not known if secondary mutations within any target genes as a result of MSH3 dysfunction change the behavior of colorectal cancer.

Mutation of *MSH3* itself can occur as a consequence of MSI-H cancers (sporadic or Lynch) due to its exon 7 coding [A_8_] microsatellite that can be subject to frameshift. This secondary mutation of *MSH3* happens in about one-third to one-half of sporadic MSI-H colorectal cancers [49,50] and perhaps less often among Lynch cancers [51]. As all of the DNA mismatch repair genes are tumor suppressors meaning that both alleles must be absent for loss of function, it is not clear from some reports if biallelic mutation occurs in all of the cancers [52]. What is not known if the additional loss of MSH3 function enhances any characteristic of an already MSI-H colorectal cancer which already has full loss of DNA mismatch repair (as in the case of *MLH1* hypermethylation, or germline mutation of *MLH1* or *MSH2,* or *PMS2*, but perhaps could enhance characteristics of germline *MSH6* mutation carriers). The addition of an *MSH3* mutation on top of hypermethylation of *MLH1*, for instance, would combine the defects for recognition and repair outlined in Table 1, conceivably influencing the ultimate behavior of the cancer. One report indicates that MSH-H colorectal cancers with secondary *MSH3* mutation demonstrates decreased wall invasiveness and aneuploidy histologically [50], but this has not been confirmed in any other study.

Among other conditions, dysfunction of MSH3 might drive pathogenesis. Cell-free extracts containing defective MutSβ can catalyze expansions and contractions at trinucleotide repeats in the absence of any DNA replication—an important concept for several neurological conditions in which trinucleotide repeat expansions are pathogenic, but without neuronal mitosis [53]. Utilizing congenic mouse models, biased expansion of the [CAG] repeat in the Huntington’s gene (which potentially causes disease) in liver and striatum occurred as a consequence of a polymorphism in the *MSH3* gene, which altered its protein levels and function [54]. Thus, *MSH3*-deficiency may be important in accentuating or initiating specific conditions even without DNA replication.

Expression levels of MSH3 in cells and tissues could be an important factor, as dysregulation of MSH3 might affect cells and tissues differently with high or low levels of MSH3. There are substantially more MutS proteins (MSH2, MSH6 and MSH3) than MutL proteins in cells, and the stability of MSH6 and MSH3 is dependent on expression of MSH2 [11]. In cells in which *MSH6* is inactivated, levels of *MSH3* transcripts increase and there is enhanced MSH3 protein stability [11]. Levels of MSH2, MSH6 and MSH3 protein, although ubiquitous in cells, vary widely in murine tissues, with some tissues expressing MSH3 at higher levels than MSH6 [55]. The varying levels of DNA mismatch repair protein expression might be a basis for functional reliance on some proteins versus others, with inactivation affecting organs differently.

Mutation and repair efficiency of I/D loops by MutSβ can vary, as it does for MutSα. The number of repeat units of the microsatellite may dictate slippage and proneness for repair, with longer lengths more likely to mutate with MutSβ deficiency [56,57,58]. Additionally, the nucleotides surrounding the microsatellites can dictate the likelihood for frameshift mutation and repair [56,57,58], and formational dynamics of trinucleotide repeat loop junctions may dictate the ability of MutSβ to bind, bend, and dissociate from DNA [59]. Thus, not only may loss of MSH3 affect specific tissues differently, but any potential target gene mutations that might be important for those tissues may depend on the dynamics of local DNA.

## 8. MSH3 is Involved in Double Strand Break (DSB) Repair

It is clear that MSH3 is a critical recognition component of the DNA mismatch repair complex MutSβ, and recognizes larger I/D loops of greater than two nucleotides (see Table 1 and Table 2). However, several lines of evidence indicate that MSH3 and MutSβ participate in double strand break (DSB) repair, unique to this DNA mismatch complex. Campregher *et al*. demonstrated with *MSH3* silencing in HCEC cells that RAD50 and MRE11 were overexpressed, indicative of DSBs [27]. Takahashi *et al*., and Park *et al*. showed that *MSH3*-deficient cells maintained higher levels of phosphorylated H2AX and 53BP1, markers of DSBs, after oxaliplatin-induced interstrand cross links over *MSH3*-proficient cells [60,61]. Additionally, van Oers *et al*. showed that murine *MSH3*-null fibroblasts developed chromatid breaks after radiation compared with *MSH3*-proficient cells, and that *MSH3*-deficient mice in a *TP53*-null background developed late onset tumors with increased loss of heterozygosity (LOH) and copy number variation (both indicative of chromosomal instability), and demonstrated EMAST [62]. These authors suggest that in contrast to an MSH2 defect in which there is a strong and dominant mismatch repair defect and only a moderate DSB repair defect, an MSH3 defect is moderate for mismatch repair as well as for DSB repair with observation of both defects in tumors [62]. Indeed, Dietlien *et al*. showed that human cells with *MSH3* mutations have a clear defect in homologous recombination repair for DSBs, making the cells dependent on non-homologous end joining (NHEJ) repair for DSBs mediated by DNA PKcs, encoded by *PRKDC* [63]. Because MSH3-deficient cells are addicted to DNA PKcs for repair of DSBs, the authors demonstrate that inhibition of DNA PKcs can induce apoptosis in *MSH3*-mutant cells, a promising therapeutic approach to target these cells [63]. Thus, MSH3 participates in both DNA mismatch repair as well as in homologous recombination repair of DSBs, making loss of MSH3 function (or the appearance of EMAST) a complex repair defect in cells.

## 9. Summary

EMAST is a biomarker for loss of MSH3 (MutSβ) function in DNA mismatch repair within cells. Loss of MSH3 can occur with mutation in MSI-H colorectal cancers, but it is not clear if the additional loss of MSH3 adds further phenotype to the cancer cells. Isolated loss of MSH3 function can occur with inflammation, directed by cytokines like IL6 to mis-localize MSH3 from the nucleus to the cytosol, allowing accumulation of mutations in nuclear DNA. Hypoxia and low pH may be other factors within colorectal cancers or non-cancer inflamed tissues to reduce *MSH3* expression. The loss of MSH3 function may do more than generate EMAST; it may contribute to aneuploidy due to its role in DSB repair.

The consequences of EMAST and MSH3 dysfunction for patient care include an association with advanced stage colorectal cancer as well as reduced survival; it is not clear what the consequences are amongst patients with non-cancer inflamed tissue other than perhaps priming the tissue towards neoplasia [30], but this is speculative. There is evidence that the approach to patient therapy for cancer may need to be modified as a result of MSH3 dysfunction [63,64], but this needs to be tested in a patient population rather than cells in culture. A comparison of chemotherapeutic agents and sensitivity between the MutS recognition complexes is listed in Table 1. Specifically, the topoisomerase I inhibitor irinotecan may be more effective in MSI-H cells [65,66,67], but this may not be due to specific MutS complex binding compared to other factors, such as *TP53* mutational status [66,67], frameshift mutation of target genes such as *MRE11* [68], or increased levels of γ-H2AX and phospho-Chk2 to stabilize cell cycle dynamics [69]. Additionally, two studies suggest that irinotecan is beneficial in patients with advanced MSI-H colorectal cancer [70,71] while three studies indicate no difference with irinotecan [72,73,74]. Irinotecan has not been examined in EMAST colorectal cancer patients.

**Figure 4:**
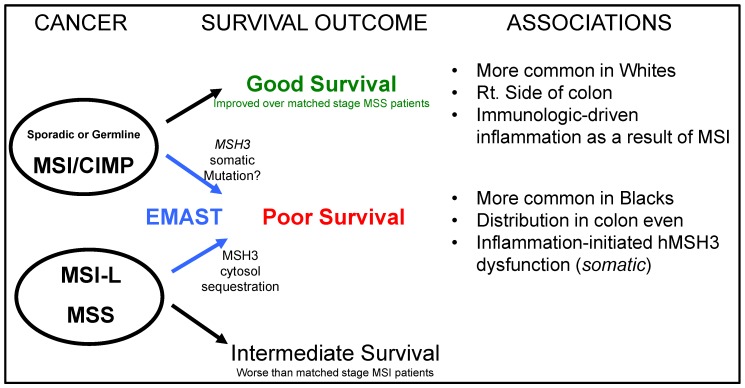
Summary diagram relating colorectal pathogenesis that may be modulated by EMAST, affecting patient outcome. Colorectal cancers can be dichotomized into MSI-H and MSS, and previously MSI-L was lumped in with MSS cancers. EMAST, the biomarker for loss of MSH3 (MutSβ function), may modify the behavior of colorectal cancer, worsening patient survival. This is in contrast to patients with MSI-H colorectal cancers with the dominant genotype of loss of DNA mismatch repair and who have good survival outcome. Among EMAST cancers, a more balanced defect between moderate loss of mismatch repair and moderate loss of repair of double strand breaks may drive the overall worse behavior. Data indicates that there are racial differences for the prevalence of MSI-H and EMAST, as well as the type of inflammation associated with each biomarker.

Although the EMAST biomarker is inclusive among MSI-H colorectal cancers due to the complete absence DNA mismatch repair, it, as a stand-alone biomarker with isolated MSH3 dysfunction, shows a different clinicopathogenic portfolio when compared to MSI-H cancers (Table 3). Unlike MSI-H colorectal cancers that generate neoantigens from frameshifted proteins that immunize the patient’s tumor and foretells an improved patient prognosis, EMAST cancers seem to develop as a consequence of inflammation (see Figure 3), modulating the baseline genomic instability of the tumor into one that is more aggressive and more likely to metastasize, and associated with poor patient survival (Figure 4). The source for the inciting inflammation for EMAST has not been investigated, but could involve the bowel contents including food debris, the microbiota and its fermentation or metabolic products, bile acids, as well as other metabolites [75,76,77]. Increased inflammation and EMAST is observed during the adenoma-carcinoma sequence [22], suggesting that the neoplasia or its morphology might perpetuate inflammation [22], and the role of immune cells needs to be examined [78]. Further exploration into the inciting and driving events that modify patient outcome as a result of EMAST and MSH3 dysfunction in colorectal cancers should yield potential approaches for primary or secondary intervention for patients [79,80].

**Table 3:** Comparison of features between MSI-H and EMAST colorectal tumors. MSS = microsatellite stable; MSI-L = microsatellite instability-low; EMAST = elevated microsatellite alterations at selected tetranucleotide repeats.

	MSI-H	EMAST	References
Genomic Instability	Microsatellite instability (MSI)	Mostly MSS and MSI-L, includes MSI-H	[16,17,18,19,49]
Germline cause	Mutation of DNA MMR gene	None known	[4,5]
Sporadic cause	*MLH1* hypermethylation	Inflammation and alteration of *MSH3*	[21,22,23,26,27,30,31]
Prevalence in sporadic CRC	~15%	Up to 60%	[9,10,16,17,20,21,22,23]
Inflammation	Crohns-like around tumor (tumor margin)	Associated with tumor nests around epithelial components	[10,21,22,23]
Immune Reaction	Neo-peptide driven; unknown but favorable	Unknown; unfavorable	[10,32]
Prognosis	Better survival; early stage	Poorer survival; later stage	[8,19,21]
Pathogenesis	Target gene mutation	Unknown; target gene mutation? Chromosomal instability?	[9,49,57,58,59]
Race	½ frequent in American Blacks	Twice frequent in American Blacks	[21,32]
Response to 5FU	Completely muted	Reduced?; not known	[7,9,10,64]

## Abbreviations

MSI: Microsatellite instability
MSS: microsatellite stable
EMAST: elevated microsatellite alterations at selected tetranucleotide repeats
CRC: colorectal cancer
MMR: DNA mismatch repair
MSH3: human MutS homolog 3
